# Editorial: Noncoding RNA-based spatiotemporal modulation and therapeutics in neuroinflammation

**DOI:** 10.3389/fimmu.2023.1276943

**Published:** 2023-09-01

**Authors:** Zhongdi Cai, Feng Gao, Jinbo Cheng, George E. Barreto, Rui Liu

**Affiliations:** ^1^ Institute of Medicinal Biotechnology, Chinese Academy of Medical Sciences and Peking Union Medical College, Beijing, China; ^2^ Department of Neuroimmunology, Henan Institute of Medical and Pharmaceutical Sciences, Zhengzhou University, Zhengzhou, China; ^3^ Center on Translational Neuroscience, College of Life & Environmental Science, Minzu University of China, Beijing, China; ^4^ Department of Biological Sciences, University of Limerick, Limerick, Ireland

**Keywords:** central nervous system disorders, drug target, neuroinflammation, neuromyelitis spectrum disorder, neuropathic pain, noncoding RNA, noncoding RNA therapy, vascular dementia

Neuroinflammation is a multifactorial process occurring in the central nervous system (CNS) that is intimately linked to temporal and spatial regulation of gene expression mediated by noncoding RNAs (ncRNAs). ncRNAs are present at high concentrations in the CNS and show specific multidimensional expression, exerting immunomodulatory effects via direct or indirect interactions with various effector proteins or other molecules to form complex networks that regulate downstream immune response pathways (1, 2). Next-generation sequencing has identified various ncRNAs dysregulated in CNS disorders, including long ncRNAs (lncRNAs), microRNAs (miRNAs), and circular RNAs (circRNAs) (3–5). Genome-wide association studies (GWAS) have also revealed numerous single nucleotide polymorphisms within lncRNAs and miRNAs (6), providing promising candidates for therapeutic targets or biomarkers for CNS disorders. This Research Topic focuses on the latest discoveries, insights, and advances in ncRNA-based signaling pathways related to novel drug targets and biomarkers in neuroinflammation.

## New developments of gene role in CNS diseases

Peripheral neuropathic pain caused by primary damage or injury to the peripheral nervous system is a complex and debilitating condition. Few treatments are currently available for neuropathic pain, partly due to incomplete understanding of the underlying mechanisms. Recent evidence has revealed dysregulated expression of lncRNA in damaged nerves, dorsal root ganglion (DRG), and spinal dorsal horn following peripheral nerve injury (7). Maruyama et al. report that lncRNA Neat1 (Neat1) is upregulated in DRG following peripheral nerve injury via Neat1-mRNA interaction-dependent and independent mechanisms, which promotes spinal neuroinflammation (microglial activation and pro-inflammatory cytokine upregulation) mediating peripheral neuropathic pain. Neat1 is causally involved in neuropathic pain and regulates spinal microglia activation through neuro-immune communication. Further investigation of Neat1’s function could lead to novel treatment options for neuropathic pain. Advances in high-throughput sequencing over the last decade have revolutionized transcriptome research, propelling drug discovery for neurological disorders (8–10). Maruyama et al. also utilize RNA sequencing to identify several key inflammatory genes involved in spinal microglia activation, including the C-C motif chemokine ligand 2 (*CCL2*) gene, a regulator of peripheral immune cell migration expressed in spinal cord astrocytes (11). Wang et al. explore the role and potential mechanisms of *CCL2* in aquaporin-4 immunoglobulin G (AQP4-IgG)-induced astrocyte injury. They show that the CCL2 level in cerebrospinal fluid from neuromyelitis spectrum disorder (NMOSD) patients is significantly higher than in other non-inflammatory neurological disease groups and that blocking astrocyte *CCL2* gene expression effectively attenuates AQP4-IgG-induced injury and reduces inflammatory cytokine release (interleukin 6 and 1 beta). The findings support that *CCL2* is involved in initiating astrocyte damage via AQP4-IgG, making it a promising candidate target for the treatment of neuroinflammatory diseases.

## ncRNA-based potential drug candidates and therapeutic perspectives

Another contribution focuses on ncRNA-based drug molecule discovery for the treatment of CNS disorders. Tilianin, the main active ingredient in the traditional Chinese medicine *Dracocephalum moldavica* L., exerts neuroprotective effects by inhibiting calcium/calmodulin-dependent protein kinase II (CaMKII)-dependent apoptotic and inflammatory pathways in dementia (12, 13). Sun et al. perform an in-depth study and elucidate that two miRNAs, namely miR-193b-3p and miR-152-3p, play a cognitive protective effect in vascular dementia (VaD), demonstrating that tilianin inhibits inflammation and apoptosis dependent on the miR-193b-3p/calmodulin (CaM) and miR-152-3p/CaMKIIα co-initiated p38 mitogen-activated protein kinase (MAPK)/nuclear factor kappa B (NF-κB) p65 and BCL2 apoptosis regulator (Bcl-2)/BCL2 associated X, apoptosis regulator (Bax)/caspase-3/poly(ADP-ribose) polymerase (PARP) pathways. These findings provide insights into the potential use of tilianin for treating VaD, highlighting its role as a promising small-molecule regulator of miRNA-associated inflammatory responses. The potential role of miRNAs in regulating neuroinflammation has been widely demonstrated (14). Zhang et al. report the functions and regulatory mechanisms of miRNAs associated with neuroinflammation-mediated CNS diseases, involving the NF-κB, MAPK, and NLR family pyrin domain containing 3 (NLRP3) signaling pathways, which are closely associated with pro-inflammatory responses. Therapeutic approaches targeting miRNAs in CNS diseases are also addressed, proposing that the combination of drug therapy and specific miRNAs could play a broader and prospective role in treatment.

Overall, this Research Topic explores the functions and molecular mechanisms of ncRNAs in immune-inflammatory responses in CNS disorders, especially neuropathic pain, NMOSD, VaD, and spinal cord brain injury. Since inflammatory ncRNAs are differentially expressed with biological regulatory effects in the peripheral circulation of CNS patients, we hope that highlighting these recent advances in ncRNA-oriented innovative pathogenesis, candidate targets, and active compounds and summarizing drug development targeting ncRNAs and neuroinflammation will motivate further research leading to diagnostic and therapeutic discovery in CNS diseases. This Research Topic was achieved by the diligent work of the Editorial Office of Frontiers in Immunology, the participating authors, and the reviewers, and we sincerely appreciate their valuable contributions to this Research Topic.

## Author contributions

ZC: Writing – original draft. FG: Writing – review & editing. JC: Writing – review & editing. GB: Writing – review & editing. RL: Conceptualization, Writing – original draft.
